# Biomechanical factors associated with patellofemoral pain in children and adolescents

**DOI:** 10.1038/s41598-024-64394-w

**Published:** 2024-07-05

**Authors:** Gerônimo J. B. Sanchis, Jeisyane A. S. do Nascimento, Rebeca de C. Santana, Vagner M. dos Santos, Vitor L. da Cunha, Sanderson J. C. de Assis, Rafael L. Cavalcanti, Thaís S. R. Guedes, Angelo G. R. da C. Oliveira, Marcello B. O. G. Guedes

**Affiliations:** 1 Federal University of Rio Grande Do Norte, Lagoa Nova, University Campus, Natal, RN 59078-900 Brazil; 2 Brazil Federal University of Minas Gerais, Belo Horizonte, Brazil; 3 Universidad de Valencia, Valencia, Spain

**Keywords:** Patellofemoral pain syndrome, Students, Knee, Biomechanics, Cross-sectional studies, Musculoskeletal system, Disease prevention, Health policy, Health services, Paediatrics, Public health, Quality of life, Bone, Bone quality and biomechanics, Physiology, Diseases, Risk factors, Signs and symptoms

## Abstract

To investigate the biomechanical factors associated with patellofemoral pain in children and adolescents. A cross-sectional, population-based study conducted in Brazil from 2019 to 2023, involving students from public schools. Adjusted prevalence ratios and their respective 95% confidence intervals for the outcome in relation to independent variables were calculated for association analysis, adopting a significance level of 5%. Out of the total of 283 students, 152 were female and 182 were aged between 16 and 18 years old. A positive association was observed between the presence of patellofemoral pain and a poor movement quality in both lower limbs (right side: *p* = 0.04 and left side: *p* = 0.04) as well as with dynamic valgus of the left lower limb (*p* < 0.01). Patellofemoral pain in children and adolescents is associated with poor movement quality in the lower limbs and dynamic valgus of the left lower limb. Actions targeting these biomechanical factors may be crucial for early diagnosis and clinical treatment of this disfunction.

## Introduction

Patellofemoral Pain (PFP) is a diffuse painful condition that affects the retropatellar and peripatellar areas of the knee^1^. Pain may appear gradually or suddenly and tends to worsen during activities such as squatting, running, ascending and descending stairs. PFP primarily affects adolescents, young active adults and elite athletes, exhibiting prevalence up to twice as high in women^2^. Among young students, PFP may directly correlate with reduced well-being, limited social engagement and sports practice, potentially resulting in kinesiophobia, deficits in dynamic balance and lower academic performance^3^.

One of the primary factors possibly associated with PFP is the muscular imbalance around the patella^4^. The ineffective force relationship among specific muscle groups could influence the severity of this condition's impact on affected individuals^5^. Moreover, the joint structure and other biomechanical factors in the lower limb might also correlate with PFP^6^.

In a meta-analysis conducted by Neal et al*.* (2019)^7^, the authors aimed to assess numerous risk factors for PFP across diverse populations, including adolescents. However, they were unable to consolidate data for an analysis of knee biomechanics and PFP in adolescents due to a lack of consistent studies on this topic. Previous studies primarily focused on kinematic variables in female adolescents, specifically analyzing the patterns of lower limb movement during the lateral step-down task (LSDT)^8^.

From this perspective, investigating kinematic patterns in adolescents with PFP, considering aspects such as midfoot mobility, dynamic valgus and ankle joint range of motion, proves to be intriguing for identifying new potential characteristics related to this syndrome. Additionally, this research utilizes low-cost instruments that are easy to apply and sensitive for use in primary healthcare settings. Furthermore, conducting studies in developing countries with unique socioeconomic conditions and different healthcare services compared to studies involving populations from developed countries is crucial. Thus, this study aims to investigate the biomechanical factors associated with PFP in school-aged individuals of both sexes in a Brazilian capital city.

## Materials and methods

### Study design

This is a cross-sectional, population-based study that aimed to investigate the biomechanical factors associated with PFP in children and adolescents from the city of Natal, Rio Grande do Norte, Brazil, from 2019 to 2023. This study was conducted following the reporting guidelines of Strobe (Strengthening the reporting of observational studies in epidemiology). The study was approved by the Research Ethics Committee of the Federal University of Rio Grande do Norte (CAAE: 07389318.1.0000.5292) and adhered to the guidelines summarized in the Declaration of Helsinki.

### Participants

We included children and adolescents aged 10 to 18 years who were enrolled in elementary/middle school, attending either morning or afternoon class sessions. The exclusion criteria adopted in this study were as follows: individuals with physical and/or mental disabilities; any health condition that could prevent the maintenance of an orthostatic position; minors without consent from their guardians; and participants aged 18 without their own consent.

Participants who reported peripatellar and/or retropatellar pain for at least one month during two or more of the following activities were classified into the PFP group: squatting, ascending and descending stairs, jumping, and prolonged sitting^5^. The study adopted the diagnostic criteria outlined by Crossley et al*.*^9^. To refine the selection criteria, a positive classification for the PFP group required symptoms persisting for at least one month, with peripatellar or retropatellar pain worsening during at least two of the aforementioned activities.

The sample was calculated based on an outcome prevalence of at least 22%, according to data from Saes and Soares^10^. To calculate the sample size, the equation {[z^2 × p (1 − p)]/ε^2} was used, where z is the confidence limit for a 5% sampling error (z = 1.96), *p* is the estimated outcome prevalence (*p* = 22%), and ε is the estimation error margin for the estimated prevalence (ε = 5%). A design effect of 1.5 was also added, resulting in a projected sample of 264 students. However, considering the need to address selection bias, loss to follow-up, and systematic errors, the sample was increased by 5%, totaling 277 individuals to be evaluated.

For school selection, a cluster sampling technique was employed using the probability proportional to size method, where the total of students served as the sample weight in each school. Thirty schools were randomly selected from the 200 municipal and state schools across the city. Subsequently, a systematic random sampling approach was utilized for student selection, based on the list obtained from the selected schools, employing a sample interval of 28.5.

### Procedures

The assessments were conducted by a team of researchers previously trained to apply the questionnaires and tests used in the study. Procedures took place at the school itself, during a brief 30-min session. During the assessments, students were barefoot and wearing their school uniforms.

To assess the movement quality, we utilized the LSDT, described by Piva et al*.*^11^. During the test, the students were instructed to stand on a 20 cm-high step with both hands on the waist, knee extended, single-leg support and the foot close to the edge of the step (two fingers distance from the edge). Then, they were asked to touch the ground with the heel (without bearing weight) and extend the leg back to the initial position. Five repetitions were performed on each lower limb.

The following parameters were used to determine LSDT score: 1–hand strategy: if the individual removes the hand from the waist to maintain balance (1 point); 2–trunk movement: if the trunk tilts (1 point); 3–flat pelvis: if one side of the pelvis rotates or tilts compared to the other side (1 point); 4–knee position: if there is medial deviation of the knee and the tibial tuberosity crosses an imaginary line from the 2nd toe (1 point), or if the knee deviates medially and the tibial tuberosity crosses the medial edge of the foot (2 points); 5–maintaining constant unilateral posture: if the subject moved away from the untested side or if the tested limb became unstable (1 point). After completing the five repetitions, the scores were categorized as follows: (a) between 0 and 1 point, good movement quality; (b) between 2 and 3 points, average movement quality; and (c) above 4 points, poor movement quality. Furthermore, individuals who exhibited medial knee displacement during the LSDT, with the tibial tuberosity crossing and surpassing the medial edge of the foot, were classified with dynamic knee valgus^12^.

To evaluate midfoot mobility, we utilized the modified Navicular Fall Test (NFT)^13^. Before the test began, a marking was made with a pen on the palpable area of the navicular tuberosity. Following this, the participants maintained a seated position while placing their foot on the ground. Then, the examiner palpated the talus bone and adjusted the foot's position using a caliper to measure the distance between the marked point and the floor. Finally, the participants stood up and the distance between the marked point and the floor was measured once more. The test values were categorized as follows: (a) between 0 and 9 mm–adequate mobility; (b) above 9.1 mm–increased mobility^14^. NFT demonstrates inter-examiner reliability with an intraclass correlation coefficient value of 0.93, confirming its usefulness and consistency in clinical assessment^15^.

To evaluate the range of motion of the ankle joint, a closed kinetic chain (CKC) was performed for the dorsiflexion movement. The procedure involved positioning the foot on a measuring tape disposed on the floor and aligned in a straight line from the wall. With the hallux positioned on the measuring tape and the knee bent against the wall, the participant executed ankle dorsiflexion within a CKC framework, sliding the foot backward while keeping the knee against the wall and the heel on the floor. Subsequently, the examiner measured the distance between the hallux and the wall. A discrepancy of 10 cm between the limbs indicated hypomobility^15^.

### Statistical analysis

The demographic characteristics were described with the use of tables, accounting for the complex sampling design. The Prevalence Rates (PR) for each variable were computed, considering the complex sampling design factors such as weights and clustering effects. To analyze the association between PFP and independent variables (gender, movement quality, dynamic valgus, midfoot mobility, and ankle range of motion), PR and their respective 95% confidence intervals (95% CI) were estimated using unadjusted outcomes from Multiple Poisson Regression with robust variance. Variables with a p-value less than 0.1 and deemed theoretically relevant were incorporated into the multiple analysis. Adjusted PR and corresponding 95% CI were then calculated. A significance level of 5% (α < 0.05) was applied. Demographic characteristics and PR analysis were conducted using SPSS (version 25), while Multiple Poisson Regression analysis was performed using Stata (Stata/IC 12.0).

## Results

A total of 344 students were contacted and 283 participated in the study. There were 61 losses, including nine due to not meeting inclusion criteria, three due to parental refusal or disapproval and 49 due to absence at the time of data collection. The prevalence of PFP among the students was 24.7% (95% CI 19–31.4) and 50.9% (95% CI: 43.6–58.1) of the sample consisted of female individuals.

Regarding NFT, we found that 21.5% (95% CI 16–28.3) and 14.8% (95% CI 10.8–19.8) of individuals showed a greater midfoot mobility in the right and left lower limbs, respectively. In the LSDT, 39.2% (95% CI 32.7–46.2) and 39.6% (95% CI 33–46.6) exhibited poor movement quality in the right and left lower limbs, respectively. Dynamic valgus was present in the right lower limb in 44.1% (95% CI 37.2–51.3) and in the left lower limb in 45.1% (95% CI 38.1–52.3) of the students. Concerning the CKC, 46.4% (95% CI 39.2–53.7) and 48.8% (95% CI 41.5–56) were classified with hypomobility in the right and left lower limbs, respectively (Table 1).

**Table 1:** Population estimates of the main categorical variables studied. Natal/RN.

Variable	Prevalence		
	n (*)	% (**)	95% CI
PFP			
Absence	210	75.3	68.6–81
Presence	73	24.7	19–31.4
Sex			
Male	131	49.1	41.9–56.4
Female	152	50.9	43.6–58.1
Age			
10 to 12 years	22	12.2	7.1–20.1
13 to 15 years	79	29.7	23.5–36.8
16 to 18 years	182	58.1	50.5–65.3
Midfoot mobility (NFT)—Right			
Adequate	223	78.5	71.7–84
Increased	60	21.5	16–28.3
Midfoot mobility (NFT)—Left			
Adequate	235	85.2	80.2–89.2
Increased	48	14.8	10.8–19.8
Movement Quality (LSDT)—Right			
Good	60	20.1	15.3–25.8
Average	103	40.7	33.4–48.5
Poor	120	39.2	32.7–46.2
Movement Quality (LSDT)—Left			
Good	39	14.8	9.9–21.5
Average	122	45.7	38.4–53.1
Poor	122	39.6	33.0–46.6
Dynamic valgus—Right			
Absent	151	55.9	48.7–62.8
Present	132	44.1	37.2–51.3
Dynamic valgus—Left			
Absent	150	54.9	47.7–61.9
Present	133	45.1	38.1–52.3
CKC—Right			
Proper	152	53.4	46.3–60.8
Hypomobility	131	46.4	39.2–53.7
CKC—Left			
Proper	142	51.2	44–58.2
Hypomobility	141	48.8	41.5–56

*PFP* patellofemoral pain, *NFT* navicular fall test, *LSDT* lateral step-down task, *CKC* closed kinetic chain; (*) Number of individuals evaluated; (**) Percentage obtained after weighting and cluster effect. Does not correspond to the same proportion of the sample.

The analysis of the independent variables showed a positive association between a poor movement quality in the LSDT and the presence of PFP on the right side, both in the unadjusted (*p* < 0.05) (PR = 1.9; 95% CI = 1.01–3.7) and adjusted (*p* = 0.04) (PR: 2.2; 95% CI 1.02–4.8) models. Similar findings were observed on the left side in the unadjusted (*p* = 0.04) (PR = 2.7; 95% CI = 1.02–7.1) and adjusted (*p* = 0.04) (PR = 2.6; 95% CI =  > 1–6.9) models. Also, there was a positive association between PFP and the presence of dynamic valgus in the left lower limb, in the unadjusted (*p* < 0.01) (PR = 1.9; 95% CI = 1.1–3.2) and adjusted (*p* < 0.01) (PR = 1.9; 95% CI = 1.1–3.1) models (Table 2).

**Table 2:** Relationship of PFP with the independent variables of the study. Sample: 283. Estimation of prevalence ratio obtained by Poisson multiple regression.

	PFP		Not adjusted		Adjusted	
	Absent	Present	*p* value	PR (95% CI)	*p* value	PR (95% CI)
Sex						
Male	104 (39.5)	27 (9.7)		1		1
Female	106 (35.9)	46 (15)	0.06	1.4 (0.9–2.2)	0.1	1.3 (0.9–2.1)
Movement Quality (LSDT)—Right						
Good	51 (16.8)	9 (3.2)		1		1
Average	83 (35.1)	20 (5.6)	0.4	1.2 (0.6–2.6)	0.3	1.3 (0.6–2.8)
Poor	85 (28)	35 (11.2)	0.05	1.9 (1.01- 3.7)	0.04	2.2 (1.02–4.8)
Movement Quality (LSDT)—Left						
Good	35 (12.5)	4 (2.3)		1		1
Average	105 (38.2)	17 (7.5)	0.5	1.3 (0.4–3.8)	0.5	1.3 (0.4–3.7
Poor	88 (26.9)	34 (12.7)	0.04	2.7 (1.02–7.1)	0.04	2.6 (> 1–6.9)
Dynamic valgus—Rigth						
Absent	120 (46.2)	31 (9.7)				–
Present	99 (33.7)	33 (10.4)	0.3	1.2 (0.7–1.8)	–	–
Dynamic valgus—Left						
Absent	130 (45.9)	20 (9.1)		1		1
Present	98 (31.7)	55 (13.4)	< 0.01	1.9 (1.1–3.2)	< 0.01	1.9 (1.1–3.1)
Midfoot mobility (NFT)—Right						
Adequate	174 (62.8)	49 (15.7)		1		–
Increased	45 (17.2)	15 (4.3)	0.6	1.1 (0.6–1.8)	–	–
Midfoot mobility (NFT)—Left						
Adequate	193 (67.2)	42 (18.1)		1	–	–
Increased	35 (10.4)	13 (4.4)	0.1	1.5 (0.8–2.6)	–	–
CKC—Right						
Proper	119 (44.6)	31 (9)		1	–	–
Hypomobility	100 (35.7)	31 (10.6)	0.5	1.1 (0.7–1.7)	–	–
CKC—Left						
Proper	119 (43.5)	22 (7.7)		1		1
Hypomobility	108 (34)	33 (14.7)	0.1	1.5 (0.9–2.4)	0.3	1.2 (0.7–2.0)

*PFP* patellofemoral pain, *LSDT* lateral step-down task, *NFT* navicular fall test.

## Discussion

This study aimed to investigate the biomechanical factors associated with PFP in children and adolescents of both sexes in a Brazilian capital city. In summary, we found a positive Association between the presence of PFP and a poor movement quality of the lower limbs, as well as with the occurrence of dynamic valgus in the left lower limb. On the other hand, no association was observed between PFP and midfoot/ankle mobility deficits. These findings provide new insights into the association between these biomechanical variables and the prevalence of PFP in young individuals, highlighting the importance of this evaluation during the school age.

Our results showed a positive association between the presence of PFP and a worse performance on the LSDT. Previous studies had already identified that individuals with poor lower limb movement quality tend to experience patellofemoral pain^16–19^. It has been discussed that the poor movement quality on LSDT could be related to muscle strength deficits, especially in hip external rotators and knee extensors. Some studies have corroborated this hypothesis, showing that these alterations could unbalance the force vectors on the patella, increasing contact pressure in the patellofemoral joint, and consequently causing pain^17,20^.

However, these studies also emphasized that one of the main biomechanical factors associated with lower limb movement quality would be the reduction in ankle dorsiflexion, unlike our findings. These discrepancies may have arisen due to differences in the biomechanical characteristics of the populations and samples used, as the aforementioned studies analyzed adult men and women, but not children and adolescents, whose biomechanical and functional patterns are still in the developmental process. Nevertheless, it's worth noting that the methodological design of this cross-sectional study does not allow us to infer whether the low level of movement quality is a cause or a consequence of knee joint pain.

Moreover, we observed an association between the presence of PFP and the occurrence of dynamic valgus, only in the left lower limb. Dynamic valgus may serve as an underlying factor in the development of insidious or chronic knee pain, such as in PFP^17^. This finding corroborates previous findings in the literature^21,22^. The presence of dynamic valgus can lead to an overload in the patellofemoral region, contributing to the pain and dysfunction observed in PFP^23^. These factors can result from abnormal load distribution in the knee region, increasing the overload in specific regions. Eventually, such changes can lead to future complications such as knee osteoarthritis^24^. Therefore, early identification of these changes in adolescents using low-cost and easy-to-use instruments can be valuable strategies for preventing these cases.

Although limb dominance was not assessed in this study, it may have contributed to this result. The left leg is predominantly the non-dominant limb and is often associated with lower capacity to generate muscle torque^25^. Greater torque capacity can help to properly maintain the patella in the joint, allowing greater energy dissipation and consequently reducing stress on the patellofemoral joint^26^. Therefore, characteristics related to the lower capacity of the musculoskeletal system to handle torques during weight-bearing activities in young individuals, especially in the non-dominant limb, may explain our findings.

We did not find any association between PFP and increased midfoot mobility. This result differs from a previous study^27^. In contrast, our study adopted a cross-sectional approach and focused on children and adolescents, not finding a positive association with the outcome in question. A potential explanation for this finding is the ongoing biological maturation. It is possible that the association between foot mobility and PFP becomes more apparent or significant in later stages of life, as movement patterns and joint loads become more established. This is because the musculoskeletal system is still developing during this stage of life. Other factors, including muscular/biomechanical compensations, neurophysiological mechanisms, psychological aspects, and environmental factors, may affect this process^28^. Therefore, the complexity of these variables requires more comprehensive studies to fully understand the underlying mechanisms of PFP in this population.

While other studies observed that ankle joint restriction or instability during closed kinetic chain dorsiflexion movement can influence movement patterns and the biomechanics of nearby joints, potentially being a risk factor for excessive dynamic valgus development and a contributor to the pain observed in PFP^28,29^, our findings do not support this assumption. It's important to note that our study focused on children and adolescents. While we did not find a positive association with the outcome, given the cross-sectional nature of our study, it's conceivable that the effects of dorsiflexion limitation could lead to long-term changes or impact biomechanical alignment during the growth phase.

Also, we did not observe an association between PFP and limited ankle dorsiflexion range of motion during CKC movement. The observed discrepancy may have been influenced by differences in the age range of the studied samples. In our study, the sample consisted of children and adolescents, unlike previous studies where the samples consisted of adults^28,29^. This hypothesis is reinforced by the fact that children and adolescents undergo constant changes, which may result in greater flexibility and adaptability compared to older individuals^30,31^. The plasticity and specific characteristics of the musculoskeletal system in each age group may lead to distinct responses to ankle dorsiflexion restrictions, contributing to the differences in results found among studies.

Among our study's limitations, it's important to acknowledge that the cross-sectional design prevents establishing causal relationships between the examined factors and the occurrence of PFP. Longitudinal or experimental approaches could provide clearer insights into temporal and causal associations. We didn't address significant kinetic variables, like knee extensor strength and lateral hip musculature or biopsychosocial factors. Furthermore, it's important to note that our study focused on children and adolescents aged between 10 and 18 years. Therefore, comparisons with other groups, such as adults, athletes, or military personnel, should be made cautiously. Further studies are recommended to explore these aspects and enhance our understanding of PFP.

In terms of the practical implications, our findings have the potential to raise awareness about the importance of musculoskeletal health care among students. Moreover, we emphasize the usefulness of clinical tests, such as the LSDT, which has shown to be a low cost assessment option with a positive association with knee joint-related outcomes. These findings may contribute to the implementation of educational and health programs aimed at improving students' health, in order to prevent and treat musculoskeletal disorders. Professionals should consider the factors associated with PFP listed here during their evaluation and treatment process in young individuals.

## Conclusions

PFP in children and adolescents is associated with poor movement quality in both lower limbs and dynamic valgus in the left (but not in the right) lower limb. Some biomechanical factors such as midfoot mobility and CKC dorsiflexion range of motion showed no association with PFP. These aspects should be considered during a clinical evaluation of this population.

### Supplementary Information

Supplementary Information.

## Data Availability

All data needed to evaluate the conclusions in the paper are present in the paper and/or the Supplementary Materials. Any additional information are available from the corresponding author.

## References

[1] Collins NJ (2018). 2018 Consensus statement on exercise therapy and physical interventions (orthoses, taping and manual therapy) to treat patellofemoral pain: Recommendations from the 5th International Patellofemoral Pain Research Retreat, Gold Coast, Australia, 2017. Br. J. Sports Med..

[2] Smith BE (2018). Incidence and prevalence of patellofemoral pain: A systematic review and meta-analysis. PLoS One..

[3] Shallan A (2023). The association between kinesiophobia and dynamic balance in patients with patellofemoral pain syndrome. Eur. Rev. Med. Pharmacol. Sci..

[4] Petersen W (2014). Patellofemoral pain syndrome. Knee Surg Sports Traumatol Arthrosc..

[5] Crossley KM, van Middelkoop M, Barton CJ, Culvenor AG (2019). Rethinking patellofemoral pain: Prevention, management and long-term consequences. Best Pract. Res. Clin. Rheumatol..

[6] Leão, L. C., Will, R. C. C., Barini, B. F., & Melo, L.. B. Síndrome da dor patelofemoral: um estudo sob ampla perspectiva. *REAS* **15**, e11144. 10.25248/reas.e11144.2022 (2022).

[7] Neal BS (2019). Risk factors for patellofemoral pain: A systematic review and meta-analysis. Br. J. Sports Med..

[8] Foschera, L. R. Kinematics of lower limbs in adolescents with and without patellofemoral pain [Dissertation]. Rio Grande do Sul/Brazil: Federal University of Santa Maria; 2021. 45 s. Post-graduation in Functional Rehabilitation of the Health Sciences Center of the Federal University of Santa Maria (UFSM).

[9] Crossley KM (2016). 2016 Patellofemoral pain consensus statement from the 4th international patellofemoral pain research retreat, Manchester. Part 1: Terminology, definitions, clinical examination, natural history, patellofemoral osteoarthritis, and patient-reported outcome m. Br. J. Sports Med..

[10] Saes MO, Soares MCF (2017). Knee pain in adolescents: prevalence, risk factors and functional impairment. Braz. J. Phys. Ther..

[11] Piva SR (2006). Reliability of measures of impairments associated with patellofemoral pain syndrome. BMC Musculoskelet. Disord..

[12] Rabin A, Kozol Z (2010). Measures of range of motion and strength among healthy women with differing quality of lower extremity movement during the lateral step-down test. J. Orthop. Sports Phys. Ther..

[13] Sabino GS, Rocha IC, Guimarães CQ, de Alcântara MA, Felicio DC (2012). Analysis of the reliability of the clinical test of navicular fall. Physical Therapy in Motion..

[14] Allen MK, Glasoe WM (2000). Metrecom measurement of navicular drop in subjects with anterior cruciate ligament injury. J. Athl. Train..

[15] Onate JA (2018). Normative functional performance values in high school athletes: The functional pre-participation evaluation project. J. Athl. Train..

[16] Rabin A, Kozol Z, Moran U, Efergan A, Geffen Y, Finestone AS (2014). Factors associated with visually assessed quality of movement during a lateral step-down test among individuals with patellofemoral pain. J. Orthop. Sports Phys. Ther..

[17] Powers CM, Witvrouw E, Davis IS, Crossley KM (2017). Evidence-based framework for a pathomechanical model of patellofemoral pain: 2017 patellofemoral pain consensus statement from the 4th International Patellofemoral Pain Research Retreat, Manchester, UK: Part 3. Br. J. Sports Med..

[18] Mansfield CJ (2022). The association and reliability of the frontal plane projection angle during the lateral step down test on knee function in patients with patellofemoral pain. Knee.

[19] Holden S, Boreham C, Doherty C, Delahunt E (2017). Two-dimensional knee valgus displacement as a predictor of patellofemoral pain in adolescent females. Scand. J. Med. Sci. Sports.

[20] Silva RLE, Pinheiro YT, Lins CAA, de Oliveira RR, Scattone SR (2019). Assessment of quality of movement during a lateral step-down test: Narrative review. J. Bodyw. Mov. Ther..

[21] Alrayani H, Herrington L, Liu A, Jones R (2023). Frontal plane projection angle predicts patellofemoral pain: Prospective study in male military cadets. Phys. Ther. Sport.

[22] Wyndow N, Collins NJ, Vicenzino B, Tucker K, Crossley KM (2018). Foot and ankle characteristics and dynamic knee valgus in individuals with patellofemoral osteoarthritis. J. Foot Ankle Res..

[23] Scholtes SA, Salsich GB (2020). Consistency of dynamic knee valgus kinematics and pain across functional tasks in females with patellofemoral pain: A cross-sectional study. Int. J. Sports Phys. Ther..

[24] Yalfani A, Ahmadi M, Asgarpoor A (2024). The effect of kinetic factors of dynamic knee valgus on patellofemoral pain: A systematic review and meta-analysis. J. Bodyw. Mov. Ther..

[25] Dye SF (2005). The pathophysiology of patellofemoral pain: A tissue homeostasis perspective. Clin. Orthop. Relat. Res..

[26] Coppack RJ, Etherington J, Wills AK (2011). The effects of exercise for the prevention of overuse anterior knee pain: A randomized controlled trial. Am. J. Sports Med..

[27] Guan Y (2021). Bilateral difference between lower limbs in children practicing laterally dominant vs. non-laterally dominant sports. Eur. J. Sport Sci..

[28] Boling MC (2009). A prospective investigation of biomechanical risk factors for patellofemoral pain syndrome: The Joint Undertaking to Monitor and Prevent ACL Injury (JUMP-ACL) cohort. Am. J. Sports Med..

[29] Lima YL (2018). The association of ankle dorsiflexion and dynamic knee valgus: A systematic review and meta-analysis. Phys. Ther. Sport.

[30] Emamvirdi M (2023). Comparing kinematic asymmetry and lateral step-down test scores in healthy, chronic ankle instability, and patellofemoral pain syndrome female basketball players: A cross-sectional study. Sci. Rep..

[31] Dotan R (2016). Children's neuromotor and muscle-functional attributes–outstanding issues. Pediatr. Exerc. Sci..

