# Supplementary material for: Biomechanical factors associated with patellofemoral pain in children and adolescents
Source: Sci Rep. 2024 Jul 5;14:15490. doi: 10.1038/s41598-024-64394-w (PMC11226601; doi:10.1038/s41598-024-64394-w)
Supplement: Supplementary file 1 — Supplementary Information. [file 41598_2024_64394_MOESM1_ESM.pdf]

SUPPLEMENTARY INFORMATION

BIOMECHANICAL FACTORS ASSOCIATED WITH PATELLOFEMORAL PAIN SYNDROME IN CHILDREN AND ADOLESCENTS

Authors: Gerônimo J B Sanchis1; Jeisyane A S de Nascimento2*; Rebeca de C Santana3; Vagner M dos Santos4; Victor L da Cunha5; Sanderson J C de Assis6; Raphael L Cavalcanti7; Thaís S R Guedes8, Angelo G R da C Oliveira9; Marcello B O G Guedes10

ADDITIONAL INFORMATION

Statement of preview and approval of the final version

We declare that all authors have viewed and approved the final version of the manuscript entitled "Biomechanical factors associated with patellofemoral pain syndrome in children and adolescents" that is being submitted. And that this article is the original work of the authors, has not received prior publication, and is not being considered for publication elsewhere. Authors' contributions:

Geronimo J B Sanchis: Documento assinado digitalmente
GERONIMO JOSE BOUZAS SANCHIS
Data: 29/09/2023 10:21:16-0300
Verifique em https://validar.iti.gov.br

Jeisyane A S Nascimento: Documento assinado digitalmente
JEISYANE ACSA SANTOS DO NASCIMENTO
Data: 27/09/2023 11:37:28-0300
Verifique em https://validar.iti.gov.br

Rebeca de C Santana: Documento assinado digitalmente
REBECA DE CASTRO SANTANA
Data: 27/09/2023 16:04:23-0300
Verifique em https://validar.iti.gov.br

Vagner M dos Santos: Documento assinado digitalmente
VAGNER MESSIAS DOS SANTOS
Data: 27/09/2023 15:23:06-0300
Verifique em https://validar.iti.gov.br

Vitor L da Cunha: Documento assinado digitalmente
VITOR LEANDRO DA CUNHA
Data: 27/09/2023 16:46:10-0300
Verifique em https://validar.iti.gov.br

Sanderson J C de Assis: Documento assinado digitalmente
SANDERSON JOSE COSTA DE ASSIS
Data: 27/09/2023 18:54:37-0300
Verifique em https://validar.iti.gov.br

Rafael L Cavalcanti: Documento assinado digitalmente
RAFAEL LIMEIRA CAVALCANTI
Data: 27/09/2023 19:38:29-0300
Verifique em https://validar.iti.gov.br

Thais S R Guedes: Documento assinado digitalmente
THAIS SOUSA RODRIGUES GUEDES
Data: 27/09/2023 12:42:27-0300
Verifique em https://validar.iti.gov.br

Angelo G R da C Oliveira: Documento assinado digitalmente
ANGELO GIUSEPPE RONCALLI DA COSTA OLIVE[illegible]
Data: 29/09/2023 10:15:09-0300
Verifique em https://validar.iti.gov.br

Marcello B O G Guedes:

Documento assinado digitalmente
MARCELLO BARBOSA OTONI GONCALVES GUED
Data: 27/09/2023 11:49:58-0300
Verifique em https://validar.iti.gov.br

Statement consent

UFRN - HOSPITAL UNIVERSITÁRIO ONOFRE LOPES DA UNIVERSIDADE

PlataForma Brasil

PARECER CONSUBSTANCIADO DO CEP

DADOS DO PROJETO DE PESQUISA

Título da Pesquisa: ANÁLISE DOS FATORES DE RISCO RELACIONADOS À DOR NO JOELHO EM ESCOLARES DO MUNICÍPIO DE NATAL/RN.

Pesquisador: Geronimo Jose Bouzas Sanchis

Área Temática:

Versão: 2

CAAE: 07389318.1.0000.5292

Instituição Proponente: Pós-Graduação em Saúde Coletiva

Patrocinador Principal: Financiamento Próprio

DADOS DO PARECER

Número do Parecer: 3.360.673

Apresentação do Projeto:

Trata-se de projeto de mestrado que visa analisar os fatores de risco relacionados a dor no joelho em escolares de ambos os gêneros, idade entre 10 e 17 anos,matriculados no ensino público fundamental e médio do município de Natal/RN.

Objetivo da Pesquisa:

Objetivo Primário:

Analisar os fatores de risco relacionados a dor no joelho em escolares de ambos os gêneros, idade entre 10 e 17 anos, matriculados no ensino público fundamental e médio do município de Natal/RN.

Objetivo Secundário:

Estimar a prevalência de dor no joelho, valgo dinâmico em escolares com idade entre 10 e 17 anos, ambos os gêneros, matriculados no ensino público fundamental e médio de Natal/RN. Associar as alterações posturais a fatores socioeconômicos, demográficos,

antropométricos, comportamentais e ergonômicos. Comparar a prevalência de dor no joelho com a idade cronológica e biológica. Testar a hipótese de que o sobrepeso/obesidade, sedentarismo, movimento inadequado se associam a dor no joelho.

Avaliação dos Riscos e Benefícios:

Riscos:

Endereço: Avenida Nilo Peçanha, 620 - Prédio Administrativo - 1º Andar - Espaço João Machado
Bairro: Petrópolis **CEP:** 59.012-300
UF: RN **Município:** NATAL
Telefone: (84)3342-5003 **Fax:** (84)3202-3941 **E-mail:** cep_huol@yahoo.com.br

Possíveis desconfortos na resposta aos formulários e questionários que serão minimizados através das seguintes providências: a avaliação e questionamento será feito individualmente com o escolar num ambiente restrito da escola.

Benefícios:

Os pais saberão se seu filho(a) apresenta sobrepeso ou obesidade; alguma alteração postural que possa trazer futuramente dores musculares ou esqueléticas. Se algum destes distúrbios estiver presente, os pais receberão orientações de prevenção e tratamento destes distúrbios e orientação para encaminhamento a um local de tratamento especializado.

Comentários e Considerações sobre a Pesquisa:

Pesquisa factível.Muito significativa para a população em estudo.

Considerações sobre os Termos de apresentação obrigatória:

Todos presentes conforme resolução vigente.

Recomendações:

Sem recomendações.

Conclusões ou Pendências e Lista de Inadequações:

Sem pendências.

Considerações Finais a critério do CEP:

1.Apresentar relatório parcial da pesquisa, semestralmente, a contar do início da mesma.

2.Apresentar relatório final da pesquisa até 30 dias após o término da mesma.

3.O CEP HUOL deverá ser informado de todos os efeitos adversos ou fatos relevantes que alterem o curso normal do estudo.

4.Quaisquer documentações encaminhadas ao CEP HUOL deverão conter junto uma Carta de Encaminhamento, em que conste o objetivo e justificativa do que esteja sendo apresentado.

5.Caso a pesquisa seja suspensa ou encerrada antes do previsto, o CEP HUOL deverá ser comunicado, estando os motivos expressos no relatório final a ser apresentado.

6.O TCLE deverá ser obtido em duas vias, uma ficará com o pesquisador e a outra com o sujeito de pesquisa.

7.Em conformidade com a Carta Circular nº. 003/2011CONEP/CNS, faz-se obrigatório a rubrica em todas as páginas do TCLE pelo sujeito de pesquisa ou seu responsável e pelo pesquisador.

UFRN - HOSPITAL UNIVERSITÁRIO ONOFRE LOPES DA UNIVERSIDADE

Plataforma Brasil

Este parecer foi elaborado baseado nos documentos abaixo relacionados:

Tipo Documento	Arquivo	Postagem	Autor	Situação
Informações Básicas do Projeto	PB_INFORMAÇÕES_BÁSICAS_DO_PROJETO_1228743.pdf	22/04/2019 10:32:42		Aceito
Cronograma	Cronograma.pdf	22/04/2019 10:31:34	Geronimo Jose Bouzas Sanchis	Aceito
Parecer Anterior	Carta_ao_CEP_HUOL.pdf	12/04/2019 20:15:09	Geronimo Jose Bouzas Sanchis	Aceito
TCLE / Termos de Assentimento / Justificativa de Ausência	TCLE_Modificado.pdf	12/04/2019 20:14:40	Geronimo Jose Bouzas Sanchis	Aceito
TCLE / Termos de Assentimento / Justificativa de Ausência	TALE.pdf	12/04/2019 15:51:58	Geronimo Jose Bouzas Sanchis	Aceito
Outros	Carta.pdf	07/02/2019 22:04:41	Geronimo Jose Bouzas Sanchis	Aceito
Outros	Folha_de_Pesquisador.pdf	01/02/2019 13:32:06	Geronimo Jose Bouzas Sanchis	Aceito
Outros	CA_Sec_Municipal.pdf	31/01/2019 13:25:05	Geronimo Jose Bouzas Sanchis	Aceito
Outros	CA_Sec_Estadual_Assinada.pdf	31/01/2019 13:18:44	Geronimo Jose Bouzas Sanchis	Aceito
Folha de Rosto	Folha_Rosto_Geronnimo.pdf	04/10/2018 18:43:00	Geronimo Jose Bouzas Sanchis	Aceito
Projeto Detalhado / Brochura Investigador	Projeto_de_Pesquisa_Geronimo_Bouzas.pdf	02/10/2018 08:44:16	Geronimo Jose Bouzas Sanchis	Aceito

Situação do Parecer:
Aprovado

Necessita Apreciação da CONEP:
Não

NATAL, 31 de Maio de 2019

Assinado por:
jose diniz junior
(Coordenador(a))

Endereço: Avenida Nilo Peçanha, 620 - Prédio Administrativo - 1º Andar - Espaço João Machado
Bairro: Petrópolis **CEP:** 59.012-300
UF: RN **Município:** NATAL
Telefone: (84)3342-5003 **Fax:** (84)3202-3941 **E-mail:** cep_huol@yahoo.com.br
